# Effects of early‐life exposure to dust mite allergen and endotoxin on the development of asthma and wheezing: The Japan Environment and Children's Study

**DOI:** 10.1002/clt2.12071

**Published:** 2021-10-13

**Authors:** Hideki Hasunuma, Yoshiko Yoda, Narumi Tokuda, Naoko Taniguchi, Yasuhiro Takeshima, Masayuki Shima, The Japan Environment and Children's Study (JECS) Group

**Affiliations:** ^1^ Department of Public Health Hyogo College of Medicine Nishinomiya Japan; ^2^ Hyogo Regional Centre for the Japan Environment and Children's Study Nishinomiya Japan; ^3^ Department of Pediatrics Hyogo College of Medicine Nishinomiya Japan

**Keywords:** asthma, dust mite, early life, endotoxin, wheeze

## Abstract

**Background:**

The effects of early‐life exposure to house dust mite allergen and endotoxin on the development of asthma are unclear in the literature. We investigated the association of early‐life exposure (0–36 months old) to house dust mite allergen and endotoxin with asthma incidence.

**Methods:**

In this novel, large‐scale, nationwide birth cohort study, 5017 participants were randomly selected from those who met the eligibility criteria. House dust was vacuum‐sampled from the children's mattresses within homes and assayed for the presence of dust mite allergen (Der 1) and endotoxin. The participants were classified into four quartiles (Q1–Q4) according to exposure levels. We defined the incidence of asthma and wheezing using questionnaires at 12, 24, and 36 months old. Odds ratios (ORs) of the incidence of asthma and wheezing by age in Der 1 and endotoxin exposure level were estimated using logistic regression.

**Results:**

The cumulative incidence rates of asthma and wheezing during 0–36 months were 10.4% and 38.1%, respectively. Significant ORs were observed in asthma onset during 12–24 months old, asthma onset during 24–36 months old, and wheezing onset during 0–12 months old in the Q4 Der 1 group. In the Q4 endotoxin group, significant positive associations between endotoxin exposure and asthma (OR 2.00, 95% confidence interval [CI]: 1.03–3.85) and wheezing (OR 1.78, 95% CI: 1.01–3.12) onset during 24–36 months old were found.

**Conclusions:**

Our results indicated that high levels of early‐life exposure to Der 1 and endotoxin in mattresses may be involved in the development of asthma.

## 1 INTRODUCTION

The prevalence of paediatric allergic diseases has recently increased in developed countries. Adoption of Western lifestyle was noted as a contributing factor, but it has not been sufficiently verified.^1^, ^2^ Western lifestyle is characterised by an increase in the time spent indoors, highly insulated airtight houses, and a more hygienic environment; in addition, it may also be characterised by changes in the pattern of exposure to house dust mite allergen and microorganisms that influence the prevalence of paediatric allergic diseases.^2^

There is sufficient scientific evidence for dust mite allergen as a factor aggravating asthma symptoms, but there is no evidence that it is a risk factor for asthma incidence.^3^, ^4^ A European large‐scale birth cohort study showed that exposure to a high concentration of allergens immediately after birth influences the development of allergic sensitisation.^5^, ^6^ Moreover, exposure to dust mite allergens is associated with dust mite sensitisation.^7^ However, intervention studies reported no associations between dust mite exposure in infancy and asthma onset at 3^8^ or 8 years of age.^9^

Previous studies suggest no consistency in the association between endotoxin, a cell wall component of gram‐negative rods and microorganism exposure marker, and asthma development. Braun‐Fahrländer et al. reported that the endotoxin concentrations in mattress dust were higher in children from farming household than in those from non‐farming household, and significantly fewer children of farmers had hay fever, asthma, and atopic sensitisation.^10^ Mendy et al. reported a positive correlation between the endotoxin concentration in dust (endotoxin concentration per gram of dust) and wheezing in infants and toddlers (0–3 years old) but an inverse correlation with asthma after they reached school age.^11^

Furthermore, recent studies in adult Americans revealed that the influence on allergic disease differs due to climate and lifestyle because of differences in exposure to endotoxin and indoor allergens such as mites and moulds.^12^, ^13^ Therefore, it is necessary to clarify the association of exposure to dust mite allergen and endotoxin with asthma development in Asian regions under different climatic regions and lifestyles in comparison with that in Western countries. Although large‐scale birth cohort studies have been conducted in Western countries, there are few reports from Asia, including Japan.

We conducted a large‐scale birth cohort study on the effects of early‐life exposure to dust mite allergen and endotoxin in mattresses on asthma and wheezing development by 0–36 months old in Japan.

## 2 METHODS

### 2.1 Participants

**FIGURE 1:**
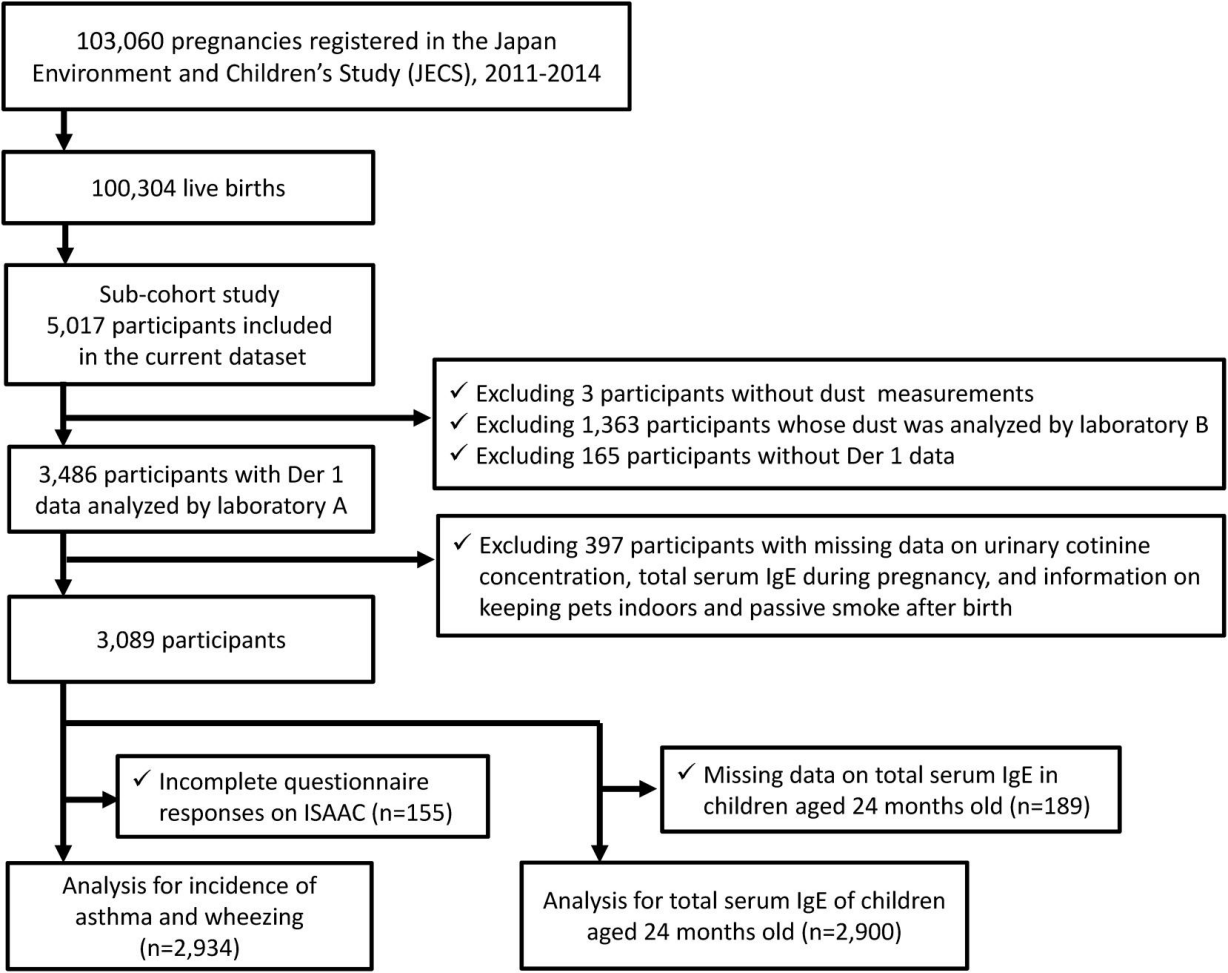
Flowchart of the selection of study participants

This is a sub‐cohort study of the Japan Environment and Children's Study (JECS). The study design and recruitment methods have been described in detail previously.^14^, ^15^ In brief, JECS is a large‐scale birth cohort study involving 100,000 pairs of parents and children in 15 nationwide regions in Japan, wherein participants were observed from the foetal period to the age of 13 years old. Participants were recruited between January 2011 and March 2014; 103,060 pregnancies were registered, of which 100,304 were live births. For the sub‐cohort study, 5017 participants were randomly selected from those who met the eligibility criteria and consented to participate in the sub‐cohort study.^16^ Data for this study were used from the dataset released in October 2019 (dataset jecs‐ta‐20190930). A schematic summarising the sample selection methodology is shown in Figure 1, and detailed study methodology is provided in Supporting information.

The JECS protocol was approved by the Institutional Review Board on Epidemiological Studies of the Ministry of the Environment (IRB number: 100406001) and by the Ethics Committees of all participating institutions. Written informed consent was obtained from all participants.

### 2.2 Questionnaire

Questionnaires were sent to the participants by post at the age of 12, 18, 24 and 36 months old, and the parents filled them out and sent them back. Those questionnaires were a modified version of ISAAC for children aged 6–7 years, with translation validation in Japanese.^17^, ^18^, ^19^ Okabe et al. investigated the validity of asthma symptoms based on ISAAC questionnaires involving 0–60‐month‐old infants by using asthma diagnosis made by allergists as criteria,^20^ wherein the sensitivity and specificity of asthma symptoms were 0.91 and 0.64 in 0–24‐month‐old children and 0.88 and 0.68 in 36–60‐month‐old children, respectively. In our study, asthma and wheezing were defined according to the definition by Okabe et al.^20^, ^21^ The incidence rates of wheezing and asthma by age (0–12, 12–24, and 24–36 months old) were defined as a ratio of participants initially judged as having wheezing/asthma at each age compared to the that of all participants.

Information on lower respiratory tract inflammation and respiratory syncytial virus (RSV) infection was obtained from the questionnaires at the age of 12, 18, and 24 months. Information concerning the presence of indoor pets and passive smoke after birth was obtained from the questionnaires at 18 months old.

### 2.3 Blood and urinal examination of mothers

Blood samples were obtained from mothers during the first trimester of pregnancy. Total serum IgE titres, assayed using ImmunoCAP (Thermo Fisher Scientific, Inc.), were analysed by an independent clinical laboratory. Mothers with high levels of total serum IgE were defined as those with a total IgE level of 214 IU/ml or above.^22^

Maternal spot urine was collected at mid‐pregnancy, and the urinary cotinine level was measured. Sample collection and measuring methods were described in detail previously.^23^ The cut‐off values for active smoking and passive smoking during pregnancy were set at 36.8 and 0.31 μg/g creatinine, respectively.^23^

### 2.4 Blood examination of children

The mothers and children visited the test site when their children were 24 months old; blood samples were obtained from children. Total serum IgE titres, assayed using ImmunoCAP (Thermo Fisher Scientific, Inc.,), were analysed by an independent clinical laboratory. As the cut‐off value of high levels of total serum IgE in 24‐month‐old children is unclear, it was set at the upper 10th percentile of the total IgE level.

### 2.5 Exposure assessment (mite allergen/endotoxin)

Trained investigators visited homes when the children were 18 months old and collected house dust from mattresses on which the children slept. A 50‐cm × 100‐cm frame exclusive for sampling was placed on the mattress, and a vacuum cleaner (Model DC61, Dyson, Japan) was run over the area in the frame for 2 min (Figure S1). The collected dust was frozen until analysis.

*Dermatophagoides pteronyssinus* (Der p 1) and *Dermatophagoides farinae* (Der f 1) in the dust were measured using an enzyme‐linked immunosorbent assay kit (Indoor Biotechnologies Ltd). Endotoxin levels in the dust were measured by kinetic chromogenic limulus amoebocyte lysate assays (Kinetic‐QCL), obtained from Lonza Japan. There is high structural similarity in the corresponding allergen between Der p 1 and Der f 1. Once sensitised, there is strong cross‐reactivity even when sensitised with either type.^24^ Thus, we used the total exposure to both mite species Der 1 (Der p 1 + Der f 1) in the analysis of the association with health outcomes.

### 2.6 Statistical analysis

Dust mite allergen and endotoxin concentrations were expressed as ‘amount per m^2^ of dust sampling area’ and ‘amount per unit mass of dust’,^25^ and Spearman's rank correlation coefficient was calculated for these exposure indices. The correlation coefficient for the ‘amount per gram of dust’ and ‘amount per m^2^ dust sampling area’ of Der 1 was 0.86, and that in endotoxin between the ‘amount per mg dust’ and ‘amount per m^2^ dust sampling area’ was 0.79, demonstrating a high correlation between these exposure indices. Thus, the ‘amount per m^2^ dust sampling area’ was analysed in the main analysis thereafter. Analysis of the ‘amount per unit mass of dust’ was performed as a sensitivity analysis. If the data were lower than those of the method detection limit, then a value of one‐half was assigned. In the analysis of the association with health outcomes, the participants were classified into the first‐to‐fourth quartile (Q1–Q4) groups.

Regarding the association between the dust mite allergen or endotoxin exposure level and health outcome, the odds ratios (ORs) of the exposure level in the Q2–Q4 groups in comparison with the Q1 group were estimated using a logistic regression model. Regarding health outcomes, the incidence rates of asthma and wheezing by age (0–12, 12–24, 24–36 and 0–36 months old) were selected for the main analysis. Secondary analysis was set to high levels of total serum IgE in children. High levels of maternal total serum IgE, active smoking/passive smoking based on the urinary cotinine level in mid‐pregnancy, passive smoke after birth, children's sex, lower respiratory tract inflammation, RSV infection, presence of indoor pets and climatic region were regarded as covariates. Climatic region was classified into three areas based on mean temperature and mean humidity (Figure S2).

A trend test was then performed to confirm the quantitative relationship of health outcomes with the exposure level. Regarding continuous variable *x* giving 1, 2, 3 and 4 points to the Q1–Q4 groups as an explanatory variable of the statistical model, the significance level, *p*‐value, of the OR of increase per unit *x* was calculated. All statistical analyses were performed using SAS ver9.4 (SAS Institute Inc.).

## 3 RESULTS

### 3.1 Participant characteristics

The characteristics of the 3089 participants included in the analysis sets are shown in Table 1. The cumulative incidence rates of asthma and wheezing were 10.4% and 38.1%, respectively. The incidence rates of asthma by age were 2.7%, 4.8%, and 3.0% at 0–12, 12–24, and 24–36 months old, respectively, and were highest at 12–24 months old. The incidence rates of wheezing by age were 20.0%, 13.6%, and 4.6%, respectively, and were highest at 0–12 months old.

**TABLE 1:** Participant characteristics

Characteristics	*n*	(%)
Sex	3089	
Boy	1576	(51.0)
Girl	1513	(49.0)
Asthma incidence	2934	
Age 0–12 months	78	(2.7)
Age 12–24 months	140	(4.8)
Age 24–36 months	88	(3.0)
Age 0–36 months	306	(10.4)
None	2628	(89.6)
Wheezing incidence	2934	
Age 0–12 months	586	(20.0)
Age 12–24 months	398	(13.6)
Age 24–36 months	134	(4.6)
Age 0–36 months	1118	(38.1)
None	1816	(61.9)
Total serum IgE level in 2‐year‐old children	2900	
High: ≥180 IU/ml	282	(9.7)
Low: <180 IU/ml	2618	(90.3)
Total serum IgE level during pregnancy	3089	
High: ≥214 IU/ml	557	(18.0)
Low: <214 IU/ml	2532	(82.0)
Urinary cotinine level in mid‐pregnancy	3089	
>36.8 μg/g‐creatinine	175	(5.7)
0.31–36.8 μg/g‐creatinine	1022	(33.1)
<0.31 μg/g‐creatinine	1892	(61.2)
Passive smoke after birth	3089	
Yes	863	(27.9)
No	2226	(72.1)
Presence of indoor pets	3089	
Yes	543	(17.6)
No	2546	(82.4)
Lower respiratory tract inflammation	3089	
Yes	471	(15.2)
No	2618	(84.8)
Respiratory syncytial virus infection	3089	
Yes	606	(19.6)
No	2483	(80.4)
Area	3089	
Cold area	1025	(33.2)
Warm low‐humidity area	1202	(38.9)
Warm high‐humidity area	862	(27.9)

The total serum IgE level was measured at 24 months old in 2900 children. The upper 10th percentile of the total serum IgE level in 24‐month‐old children was 180 IU/ml, and the serum IgE level was high (≥180 IU/ml) at 9.7% and low (<180 IU/ml) at 90.3%. High levels of maternal serum IgE were detected in 18.0%, and the rates of maternal active smoking (36.8 μg/g‐creatinine or higher) and passive smoking (0.31–36.8 μg/g‐creatinine) based on the urinary creatinine level were 5.7% and 33.1%, respectively.

### 3.2 Mite allergen and endotoxin

The levels of dust mite allergen and endotoxin in dust are shown in Table 2. The geometric means of Der 1 per unit area and per gram dust determined in the analysis were 64.95 μg/m^2^ and 3.89 μg/g, respectively. The geometric means of endotoxin were 2.71 × 10^5^ EU/m^2^ and 16.0 EU/mg, respectively.

**TABLE 2:** Concentration of dust mite allergen and endotoxin

	*N*	GM	25th	50th	75th
Der1 (Der p 1 + Der f 1)					
Der 1, μg/m^2^	3089	64.95	22.52	54.07	158.76
Der 1, μg/g	3089	3.89	1.57	3.17	8.37
Endotoxin					
Endotoxin, EU/m^2^ × 10^5^	2941	2.71	1.51	2.84	5.02
Endotoxin, EU/mg	2941	16.0	9.80	16.0	25.0

Abbreviations: Der p 1, *Dermatophagoides pteronyssinus*; Der f 1, *Dermatophagoides farinae*; EU, endotoxin unit, GM, geometric mean.

### 3.3 Association of mite allergen with the incidence of asthma or wheezing

**FIGURE 2:**
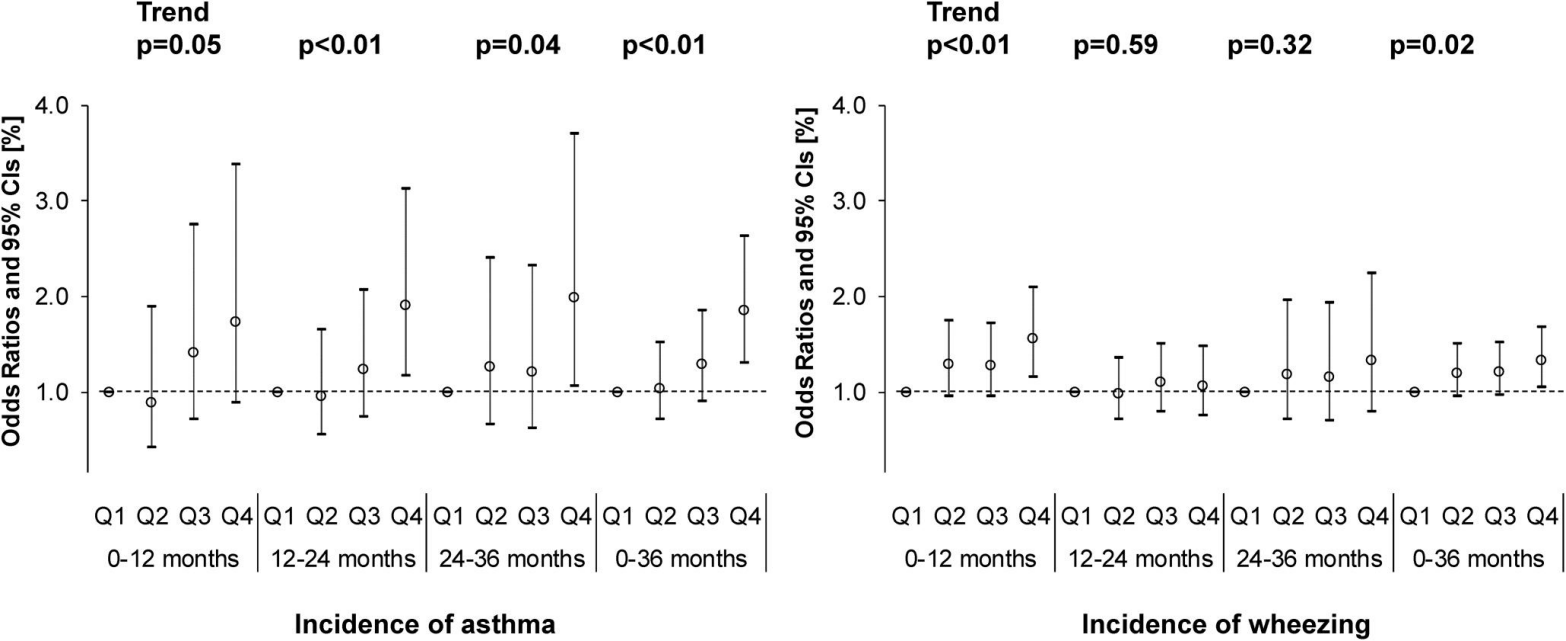
Associations among dust mite allergen, Der 1 (μg/m^2^), and incidence of asthma or wheezing using a logistic regression model adjusted for high levels of maternal total serum IgE, active smoking/passive smoking based on the urinary cotinine level in mid‐pregnancy, passive smoke after birth, children's sex, lower respiratory inflammation, respiratory syncytial virus infection, presence of indoor pets and climatic region

The adjusted ORs of dust mite allergen exposure level Der 1 in the Q2–Q4 groups in comparison with the Q1 group are shown in Figure 2. The ORs of the cumulative incidence rates of asthma and wheezing were significantly higher in the Q4 group during 0–36 months old (OR: 1.85 [95% CI: 1.30–2.63] and OR: 1.33 [95% CI: 1.05–1.67], respectively). The ORs in the Q2 and Q3 groups were not significant. A significant trend relationship with Der 1 was noted in the cumulative incidence rates of asthma and wheezing (Figure 2).

By onset age, a significant OR was noted in asthma in the Q4 group at 12–24 months old (OR 1.91, 95% CI: 1.17–3.12) and 24–36 months old (OR 1.99, 95% CI: 1.07–3.70). Similarly, a significant OR was noted in wheezing in the Q4 group during 0–12 months old (OR 1.55, 95% CI: 1.15–2.10). A significant trend was noted at all the aforementioned onset ages.

### 3.4 Association of endotoxin with the incidence of asthma or wheezing

**FIGURE 3:**
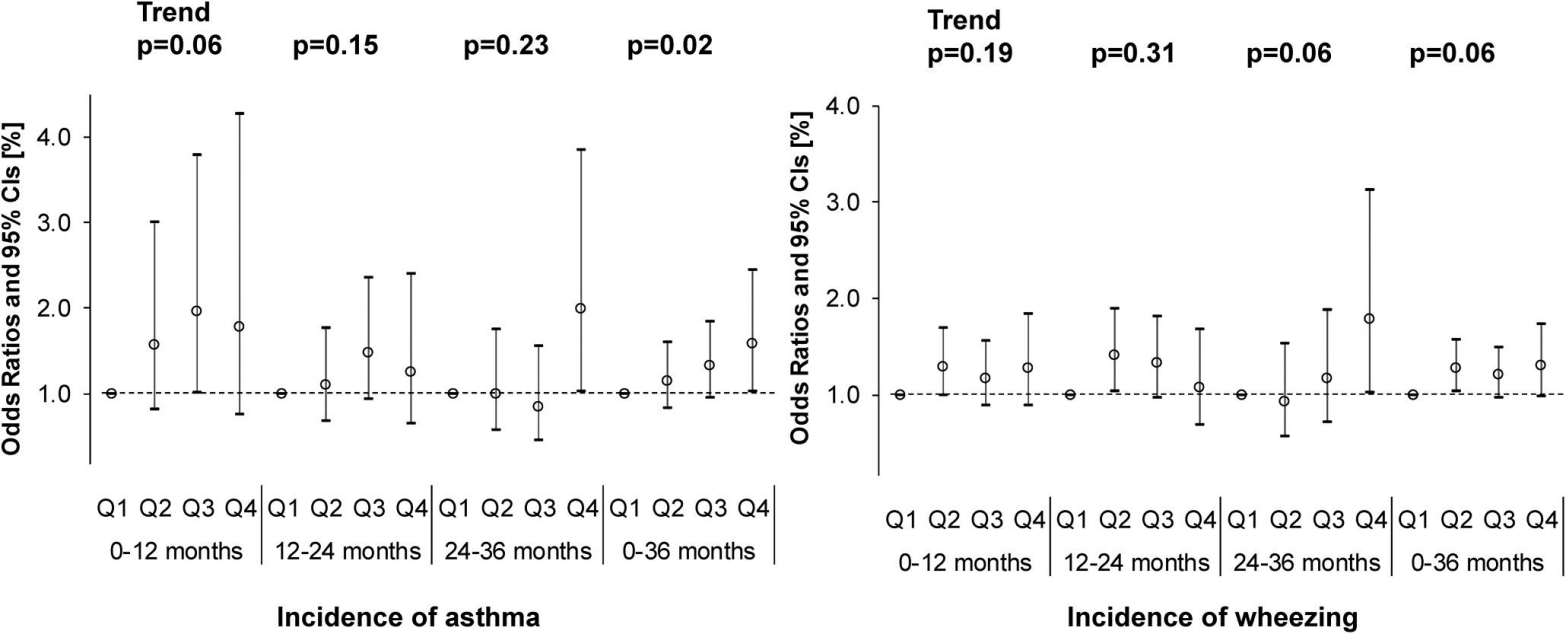
Associations between endotoxin (EU/m^2^) and the incidence of asthma or wheezing using a logistic regression model adjusted for high levels of maternal total serum IgE, active smoking/passive smoking based on the urinary cotinine level in mid‐pregnancy, passive smoke after birth, children's sex, lower respiratory inflammation, respiratory syncytial virus infection, presence of indoor pets, and climatic region

The adjusted ORs of the endotoxin exposure level are shown in Figure 3. The adjusted OR of the cumulative incidence rate of asthma during 0–36 months old was 1.59 (95% CI: 1.03–2.44) in the Q4 group of the endotoxin exposure level, indicating significance. The OR (95% CI) of the cumulative incidence rate of wheezing during 0–36 months old was significant in the Q2 group of the endotoxin exposure level (OR: 1.28 [95% CI: 1.04–1.57]). In the cumulative incidence rate of asthma, a significant trend relationship with the exposure level was noted (Figure 3).

By onset age, the ORs for the onset of asthma (OR 2.00, 95% CI: 1.03–3.85) and wheezing (OR 1.78, 95% CI: 1.01–3.12) were the highest and significant in the Q4 group during 24–36 months old.

### 3.5 Association of mite allergen or endotoxin with high levels of total serum IgE

**FIGURE 4:**
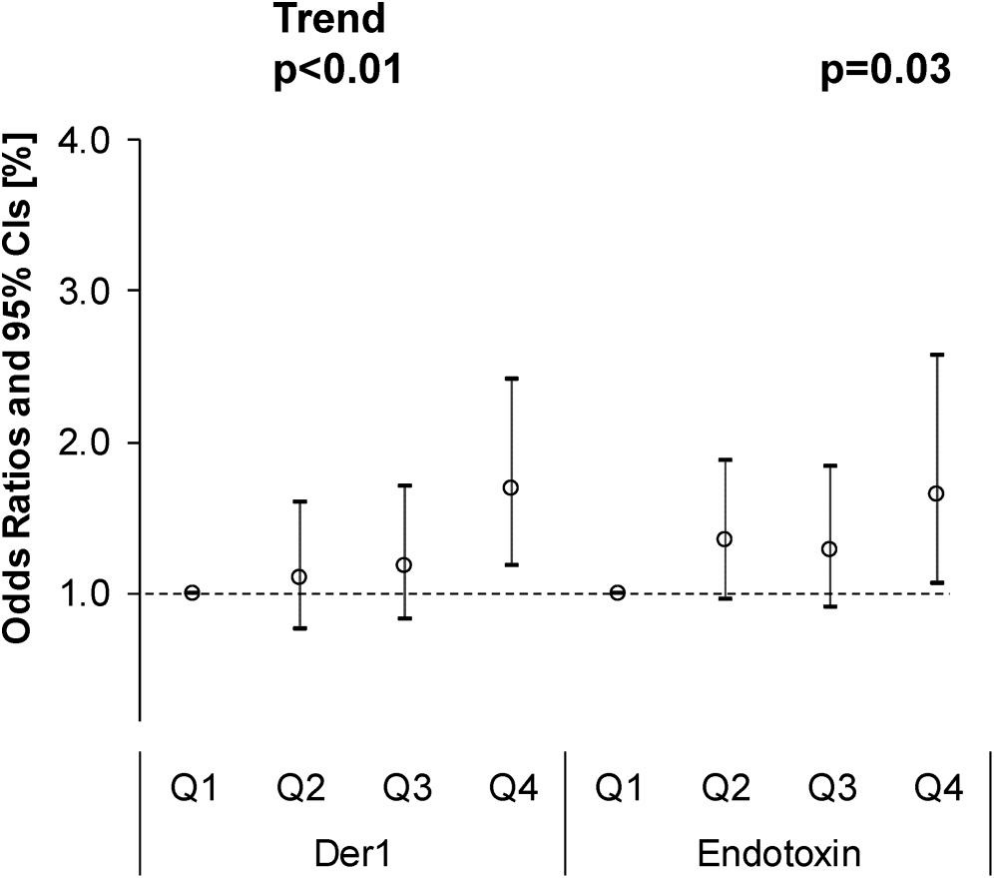
Associations between dust mite allergen, Der 1 (μg/m^2^), or endotoxin (EU/m^2^) and high serum IgE levels in children aged 24 months old using a logistic regression model adjusted for high levels of maternal total serum IgE, active smoking/passive smoking based on the urinary cotinine level in mid‐pregnancy, passive smoke after birth, children's sex, lower respiratory inflammation, respiratory syncytial virus infection, presence of indoor pets, and climatic region

The adjusted ORs for high levels of total serum IgE at 24 months old in the Q2–Q4 groups of the dust mite allergen exposure level and endotoxin exposure level in comparison with the Q1 group are shown in Figure 4. A significant OR (95% CI) was noted in both dust mite allergen and endotoxin exposure levels in the Q4 group in comparison with the Q1 group, OR: 1.69 (95% CI: 1.18–2.42) and OR: 1.66 (95% CI: 1.07–2.57), respectively, but the OR was not significant in the Q2 or Q3 group. A significant trend was noted for both dust mite allergen and endotoxin exposure levels. Results of sensitivity analysis of the ‘amount per unit mass of dust’ are shown in Figures S3–S5.

## 4 DISCUSSION

In this study, the risks of the cumulative incidence rates of asthma and wheezing increased during 0–36 months old in children with a high level of dust mite allergen exposure in bed dust. Regarding the endotoxin exposure level, a significant positive OR was occasionally noted in the onset of asthma and wheezing in the high exposure group. In the simultaneous exposure to allergen, microorganisms, fungi, and air pollutants, these were found to interact with each other and promote or prevent allergic disease.^26^ As dust mite allergen and endotoxin were simultaneously investigated in only limited epidemiological studies, these are individually discussed below.

Wahn et al. showed a dose‐response relationship between indoor allergen exposure and sensitisation during the first 3 years of life.^27^ In an integrated analysis of five geographically different European birth cohort studies, an association was noted between dust mite allergen exposure (2–6 months old) and sensitisation to house dust mite (≤6 years old), but no association was observed with an increase in the risk of asthma (≤6 years old).^6^ These findings are partially consistent with our results. In our study, the association of an increase in the risk of high levels of total serum IgE with dust mite allergen exposure was observed in the Q4 group and the OR was 1.69 (95% CI: 1.18–2.42). In addition, a trend was observed, suggesting an association between dust mite allergen exposure and sensitisation with allergen. On the contrary, no increase in the asthma risk was noted in the European cohorts, but a trend was observed between dust mite allergen exposure and the cumulative incidence rates of asthma and wheezing in the present study. This may have been due to differences in the dust mite allergen sampling point and the difference in the unit of Der 1 (μg/m^2^, μg/g).

Regarding the sampling point, dust on the floor of the children's bedroom was collected in the European cohort studies, whereas dust mite allergen in bed dust was evaluated in our study. The dust mite allergen concentration is higher in bed dust than that in dust on the bedroom floor and the dust mite allergen exposure level in children was also higher.^25^ Although it is necessary to pay attention to a simple comparison of the Der 1 concentration, the geometric mean was within a range of 0.06–0.97 μg/g in the European cohort studies, but the Der 1 concentration was higher at 3.89 μg/g in our study performed in Japan. Regarding the second point, the difference in the unit of Der 1 level, μg/g was used in the European cohort studies, whereas it was measured as μg/m^2^ in our study. The risk of mite sensitisation markedly increased when the Der 1 level exceeded 2 μg/g.^28^ In the present study, based on analysis of the amount per gram dust (unit: μg/g), no significant increase in total IgE levels was noted in the Q3 (3.17–8.37 μg/g) or Q4 (8.37 μg/g or higher) groups (Figure S5), but in the evaluation of the amount per dust sampling area, a marked increase in total IgE levels was noted in the Q4 group (158.76 μg/m^2^ or larger) (Figure 2), suggesting that the evaluation of the amount per dust sampling area is more important.

In the analyses by onset age, we observed the significant associations between dust mite allergen and the development of wheezing during 0–12 months old and asthma during 12–24 and 24–36 months old. The most important steps towards the development of mature systematic immune responses are taken in the first 1–2 years of life.^5^, ^29^ On the contrary, in the German Multicentre Allergy Study, a strong influence of viral infections in the first years of life on the development of asthma was reported,^30^ whereas the influence of sensitisation to dust mite became significant at 3 years of age.^31^ These findings suggest that the effects of mite allergen exposure to the development of asthma may differ by age. In our data, the developments of wheezing and asthma were also significantly associated with lower respiratory tract inflammation during 0–12 months old (OR: 12.49 [95% CI: 8.14–19.15] and OR: 7.70 [95% CI: 4.47–13.30]), respectively (Figure S6), and the effects of infection in the first years of life were considerably strong. However, after adjusting for those effects, the associations between dust mite allergen and the development of wheezing and asthma remained significant.

Previous studies have lacked consistency in the association between endotoxin and asthma. For example, a cohort study was performed in an urban area of Boston involving children with parents having a medical history of atopy, in which simultaneous exposure to dust mite allergen (in the children's bed) and endotoxin (in family room dust) was investigated until 2–3 months after birth, and the associations with asthma, wheezing, and atopy were surveyed until 7 years old. Endotoxin exposure was associated with an increase in the risk of asthma at 7 years old and wheezing at 1–7 years old (no change in the risk due to children's age with wheezing).^25^ Braun‐Fahrländer et al. reported that the endotoxin concentrations in mattress dust were higher in children of farming household than in those of non‐farming household, and significantly fewer children of farmers had hay fever, asthma, and atopic sensitisation.^10^ In a meta‐analysis of 19 studies involving infants, the OR of wheezing in the high endotoxin exposure group (endotoxin concentration per gram of dust) compared to that in the low exposure group was 1.48 (95% CI: 1.10–1.98), demonstrating a positive correlation, whereas the OR of asthma after the participants reached school age was 0.82 (95% CI: 0.67–0.97), demonstrating an inverse correlation.^11^ Shamosollahi et al. systematically reviewed endotoxin exposure and health influence and discussed that ‘early life exposure to endotoxin at the environmental level induces respiratory symptoms, including wheezing, because of airway inflammation’. They reported that early‐life environmental exposure to endotoxin may induce inflammatory reaction, but continuous exposure to endotoxin may activate immunity in healthy individuals and prevent the later onset of asthma.^32^

In our study, significantly positive ORs of asthma and wheezing onsets were occasionally noted in the Q2–Q4 groups of the endotoxin exposure level in comparison with the Q1 group, supporting the possibility that environmental exposure to endotoxin in early life induces an inflammatory reaction. This finding is consistent with the results of the study in the urban area of Boston, the meta‐analysis, and the systematic review. The geometric mean endotoxin concentration was 15.5 EU/mg in a survey conducted in the United States (*n* = 6953), and it was 2.5–76 EU/mg in other surveys performed in US inner cities,^33^ whereas that in dust in the present study was 16.0 EU/mg, which is within a range similar to that seen in the United States.

The main strength of this study was that this is the first large‐scale birth cohort study wherein an environmental measurement for both dust mite allergen and endotoxin and health influence were evaluated using standardised methods involving approximately 3000 infants. The cumulative incidence rate of wheezing and asthma in early life could be evaluated by conducting annual questionnaire surveys. However, this study has the following limitations. First, dust mite allergen and endotoxin exposure were evaluated only at 18 months old. Exposure to high concentrations of allergens immediately after birth strongly influences the advancement of paediatric allergic disease.^5^, ^6^ Samples were not collected immediately after birth in our study, but dust was collected at the age of 18 months old. The association with allergic disease over school age after growth will be investigated in a follow‐up survey. Second, asthma symptoms were investigated using self‐administered questionnaires filled out by mothers. However, a validation study of questionnaires that followed the diagnostic criteria of asthma used by allergists reported that the sensitivity and specificity of asthma symptoms were 0.91 and 0.64, respectively, in 0–24‐month‐old children.^20^ Third, total IgE levels at 24 months old alone were used as the cut‐off for outcome evaluation of high serum IgE levels, and the specific IgE level could not be used. Soto‐Quiros et al. reported that higher levels of specific IgE to dust mite allergen were correlated significantly with total IgE among school children.^34^ The associations with specific IgE and with changes accompanying growth remain to be investigated.

As the evaluation was performed for up to 3 years in our study, the risk (or preventive effect) of dust mite or endotoxin on the development of asthma at school age will be investigated through a follow‐up survey in the future.

## 5 CONCLUSION

Early‐life exposure to dust mite allergen was associated with the onset of wheezing during the age of 0–12 months old and asthma during the age of 12–36 months old. Early‐life exposure to endotoxin was also associated with the onset of wheezing and asthma during the age of 24–36 months old. Our results indicated that high levels of early‐life exposure to dust mite allergen and endotoxin may be involved in the development of asthma.

## CONFLICT OF INTEREST

The authors have no conflict of interest to declare.

## Supporting information

Supplementary Material 1

Supplementary Material 2
