# Supplementary material for: Effects of early‐life exposure to dust mite allergen and endotoxin on the development of asthma and wheezing: The Japan Environment and Children's Study
Source: Clin Transl Allergy. 2021 Oct 13;11(8):e12071. doi: 10.1002/clt2.12071 (PMC8514641; doi:10.1002/clt2.12071)
Supplement: Supplementary file 1 — Supplementary Material 1 [file CLT2-11-e12071-s002.docx]

**Supporting Information**

**Effects of early-life exposure to dust mite allergen and endotoxin on the development of asthma and wheezing: the Japan Environment and Children’s Study**

# Figures

**Supp Info Figure 1.**

**House dust collection method. A 50-cm × 100-cm frame exclusive for sampling was placed on the mattress on which the children slept, and a vacuum cleaner was moved over the area in the frame for 2 minutes.**

**Supp Info Figure 2.**

**The regions were classified into three climatic areas based on mean temperature and mean humidity: cold area, warm low-humidity area, and warm high-humidity area.**

**Trend**

**p=0.23 p=0.02 p=0.11 p<0.01**

**Trend**

**p=0.04 p=0.26 p=0.86 p=0.07**

**Supp Info Figure 3.**

**Associations among dust mite allergen Der 1 [µg/g] and incidence of asthma or wheezing using a logistic regression model adjusted for high levels of maternal total serum IgE, active smoking/passive smoking based on the urinary cotinine level in mid-pregnancy, passive smoke after birth, children’s sex, lower respiratory inflammation, RSV infection, presence of indoor pets, and climatic region.**

**Trend**

**p=0.03 p=0.17 p=0.60 p=0.11**

**Trend**

**p=0.14 p=0.06 p=0.44 p=0.03**

**Supp Info Figure 4.**

**Associations among endotoxin [EU/mg] and incidence of asthma or wheezing using a logistic regression model adjusted for high levels of maternal total serum IgE, active smoking/passive smoking based on the urinary cotinine level in mid-pregnancy, passive smoke after birth, children’s sex, lower respiratory inflammation, RSV infection, presence of indoor pets, and climatic region.**

**Trend**

**p=0.06 p<0.01**

**Supp Info Figure 5.**

**Associations among dust mite allergen Der 1 [µg/g], or endotoxin [EU/mg] and high levels of serum IgE in children aged 24 months old using a logistic regression model adjusted for high levels of maternal total serum IgE, active smoking/passive smoking based on the urinary cotinine level in mid-pregnancy, passive smoke after birth, children’s sex, lower respiratory inflammation, RSV infection, presence of indoor pets, and climatic region.**

**Supp Info Figure 6.**

**Adjusted odds ratios of incidence of asthma or wheezing associated with lower respiratory tract inflammation (LRI) or RSV infection using a logistic regression model adjusted for high levels of maternal total serum IgE, active smoking/passive smoking based on the urinary cotinine level in mid-pregnancy, passive smoke after birth, children’s sex, dust mite allergen Der 1, presence of indoor pets, and climatic region.**
