# Supplementary material for: Effects of early‐life exposure to dust mite allergen and endotoxin on the development of asthma and wheezing: The Japan Environment and Children's Study
Source: Clin Transl Allergy. 2021 Oct 13;11(8):e12071. doi: 10.1002/clt2.12071 (PMC8514641; doi:10.1002/clt2.12071)
Supplement: Supplementary file 2 — Supplementary Material 2 [file CLT2-11-e12071-s001.docx]

**Supporting Information**

**Effects of early-life exposure to dust mite allergen and endotoxin on the development of asthma and wheezing: the Japan Environment and Children’s Study**

# Methods

# Questionnaire

The cumulative incidence rate of wheezing (0-36 months old) was calculated as the total reported number of cases of wheezing. Wheezing was defined as a ‘yes’ answer to any of the following questions at the age of 12, 24, or 36 months old: ‘Has your child ever had wheezing or whistling in the chest at any time in the past?’ and ‘Has your child had wheezing or whistling in the chest in the past 12 months?’ The cumulative incidence rate of asthma (0-36 months old) was calculated as the total reported number of cases of asthma. Asthma was defined when a child answered ‘yes’ to the following question at the age of 12, 24, or 36 months old: ‘Immune system disorder diagnosed by doctor’.

# Sample selection methodology

Among a total of 5,017 children who were aged 18 months old, met the eligibility criteria, and consented to the Sub-Cohort study, we selected 5,014 children; house dust samples were collected from their mattress using a vacuum cleaner. Analysis of the samples for mite allergen and endotoxin levels differed depending on the timing due to the national bidding system, and it was performed by Company A (3,651 participants) during April 2015-January 2016 and by Company B (1,363 participants) during December 2016-March 2017. The analysis performed by Company A revealed that 3,486 participants had an effective dust mite allergen concentration; among them, 397 were excluded due to missing data on their urinary cotinine level and total serum IgE level during pregnancy, and information on the presence of indoor pets and passive smoke after birth was not collected from them. Therefore, 3,089 participants were considered in the analysis set. Regarding the analysis of the development of asthma and wheezing, we excluded 155 participants because they did not provide responses to the questionnaire on ISAAC at the age of 12, 24, or 36 months old. Finally, 2,934 participants were designated as the analysis set. Regarding the analysis of total serum IgE levels at the age of 24 months old, 2,900 participants were designated as the analysis set after excluding 189 participants in whom total serum IgE levels could not be measured.
